# Effect of Carvacrol, TRP Channels Modulator, on Cardiac Electrical Activity

**DOI:** 10.1155/2020/6456805

**Published:** 2020-04-06

**Authors:** Mantė Almanaitytė, Jonas Jurevičius, Regina Mačianskienė

**Affiliations:** Institute of Cardiology, Lithuanian University of Health Sciences, Kaunas, Lithuania

## Abstract

Despite the wide application of carvacrol (CAR) in medicines, dietary supplements, and foods, there is still insufficient electrophysiological data on the mechanisms of action of CAR, particularly with regard to heart function. Therefore, in this study, we attempted to elucidate whether CAR, whose inhibitory effect on both cardiac and vascular TRPM7 and L-type Ca^2+^ currents has been demonstrated previously, could modify cardiac electrical activity. We used a combination of optical mapping and microelectrode techniques to track the action potentials (APs) and the spread of electrical activity in a Langendorff-perfused rabbit heart model during atrial/endo/epicardial pacing. Simultaneously, ECG recordings were acquired. Because human trials on CAR are still lacking, we tested the action of CAR on human ventricular preparations obtained from explanted hearts. Activation time (AT), AP duration (APD), and conduction velocity maps were constructed. We demonstrated that at a low concentration (10 *μ*M) of CAR, only marginal changes in the AP parameters were observed. At higher concentrations (≥100 *μ*M), a decrease in AP upstroke velocity (*dV*/*dt*_max_), suggesting inhibition of Na^+^ current, and APD (at 50 and 90% repolarization) was detected; also slowing in the spread of electrical signals via the atrioventricular node was observed, suggesting impaired functioning of Ca^2+^ channels. In addition, a decrease in the T-wave amplitude was seen on the ECG, suggesting an impaired repolarization process. Nevertheless, those changes occurred without a significant impact on the resting membrane potential and were reversible. We suggest that CAR might play a role in modulating cardiac electrical activity at high concentrations.

## 1. Introduction

Carvacrol (CAR) is a natural monocyclic monoterpenoid (2-methyl-5-isopropylphenol), a component of thyme oil, that possesses several biological activities, such as antioxidant [1], anti-inflammatory [2], antimicrobial [3], local anaesthetic, and analgesic [4] effects (for review see [5]). In addition, CAR has been approved by the Council of Europe [6] and by the Food and Drug Administration (FDA) [7] for use in food as a flavouring agent (i.e., in sweets and beverages) and has been suggested as a dietary supplement.

Despite the wide application of CAR in medicine, in clinical dentistry, and in drinks and foods, there is still insufficient data on the mechanisms of action of CAR, particularly in the heart. In the last decade, a growing number of studies have investigated the physicochemical properties, pharmacokinetic profiles, and/or activity-structure relationships of CAR [8, 9], and numerous beneficial bioactivities of the compound have been demonstrated in cells and animals, i.e., antiallergic, anticancerogenic, hepatoprotective, gastroprotective, neuroprotective, and cardioprotective properties (for review see [5, 10]). In contrast, a very recent study revealed the side effects and cancerogenic properties of CAR at higher doses [11]. The authors uncovered CAR-induced toxic effects in both *in vivo* and *in vitro* experiments in cancer and in normal cells.

Previously, the detailed ionic mechanism of CAR action in single cells was elucidated. The reversible inhibitory effect of CAR on neuronal voltage-gated Na^+^ current (*I*_Na_) [12] and cardiac L-type Ca^2+^ current (*I*_CaL_) was demonstrated in isolated canine and human ventricular cardiomyocytes [13] and in rat mesenteric cells [14]. It is now also known that CAR is able to inhibit the mammalian transient receptor potential (TRP) melastatin-7 channels (TRPM7) expressed in HEK cells and native Drosophila TRP-like channels (TRPL) [15], as well as TRPM7-like native currents in human atrial cardiomyocytes [16], mouse hippocampal neurons [17], and rat mesenteric arteries [14]. In addition, CAR can activate TRP channels, such as vanilloid-3 (TRPV3) and ankyrin-1 (TRPA1) [18] as well as canonical-1 (TRPC1), C3, and C6 [14].

At the same time, the mechanisms of action of CAR in a whole heart and/or a heart tissue preparation, which retain intact tissue architecture and properties, thus allowing a comprehensive analysis of cardiac electrophysiology in a nearly *in vivo* setting, have not been completely elucidated. Still, the clinically relevant concentrations of the compound remain unclear in terms of safety of use and efficacy for cardioprotection, because many medicines, including natural products from plants, have side effects at high concentrations. In addition, the mechanism of electrical wave propagation in hearts pretreated with CAR has not yet been evaluated. Our study is the first to examine the extent to which clinically relevant concentrations of CAR might affect various parameters of the action potential (AP) and the spread of electrical activity in the heart.

## 2. Methods

### 2.1. Ethics

The study was carried out in accordance with the guiding principles of the European community outlined in the Declaration of Helsinki. Experiments on New Zealand white rabbits were approved by the State Food and *Veterinary* Service of the Republic of Lithuania (No. G2-34, 24 September 2015), and experiments on explanted human hearts were approved by the Ethics Committee of Biomedical Research of Kaunas Region, Lithuania (No. 2R-1344 (2.6-1), 23 February 2018).

### 2.2. Preparation of the Rabbit Heart

New Zealand white rabbits (*n* = 9) of either sex (~3 kg) were used, and the methods have been detailed previously [19]. Briefly, after intraperitoneal injection of xylazine (10 mg/kg) and heparin (1000 U/kg), intravenous injection of ketamine (10 mg/kg) was performed. Then, thoracotomy was performed, and the heart was quickly excised, cannulated through the aorta and attached to a Langendorff-perfusion system. The perfusion was carried out under constant pressure (~80 mmHg) at 37 ± 0.2°C with an oxygenated Tyrode solution (in mM: 135 NaCl, 5.4 KCl, 1.8 CaCl_2_, 0.9 MgCl_2_, 0.33 NaH_2_PO_4_, 10 glucose, and 10 HEPES; pH 7.4). After ~30 min, the perfusion was switched to a recirculation mode, and blebbistatin (10-20 *μ*M), which is an inhibitor of myosin ATPase activity and eliminates actomyosin motility, was added to stop mechanical contractions and immobilize the heart without affecting electrical recordings. After stabilization, near-infrared (NIR) voltage-sensitive dye (VSD), di-4-ANBDQBS, was added into the perfusate at a final concentration of 3 *μ*M. CAR was added to the perfusate for the final concentrations of 10, 30, 100, 300, and 1000 *μ*M. Electrical activity of the heart was obtained after 5 min of exposure to each drug concentration.

Endocardial pacing was performed via a bipolar silver electrode inserted into the left ventricular (LV) cavity close to the apex. Atrial and epicardial pacing was performed via bipolar hook electrodes embedded in the right atrium and LV epicardial surface, respectively. The heart was continuously electrically stimulated for a 300 ms period, with a 2 ms pulse width set at twice the diastolic threshold.

### 2.3. LV Wedge Preparation from Explanted Human Heart

We used human ventricular tissues prepared from explanted hearts (*n* = 3; males aged 54.3 ± 2.3 years), which were removed during heart surgery from patients undergoing heart transplantation, which were provided by the hospital of Lithuanian University of Health Sciences (LUHS) for research purposes. Informed consent was obtained before cardiac surgery. After explantation, the hearts were transported from the hospital to the laboratory in cold cardioplegic solution (in mM: 110 NaCl, 16 KCl, 1.2 CaCl_2_, 16 MgCl_2_, 5 glucose, and 10 HEPES; pH 7.4 adjusted with NaOH). A portion of the LV wall was excised together with its left anterior descending coronary artery (LAD), which was cannulated. Small leaking branches at the edges of the preparation were ligated. Then, preparation was mounted on a Langendorff apparatus and perfused through the LAD. CAR was added to the perfusate for a final concentration of 100 *μ*M. Endocardial pacing was performed via a bipolar silver hook electrode embedded in the LV endocardial surface. The preparation was continuously electrically stimulated for a 1000 ms period, with a 2 ms pulse width set at twice the diastolic threshold.

### 2.4. Microelectrode Recordings

Electrical APs were recorded using glass microelectrodes (filled with 3 M KCl) with long tips (resistance ~30-50 M*Ω*), which were inserted in the LV wall from the epicardial surface [19]. The recorded APs were amplified and digitized by the 16-channel PowerLab system (ADInstruments, Oxford, UK) at a frequency of 20 kHz. The data were recorded and analysed using the LabChart8 Pro software.

### 2.5. Optical Recordings

The optical mapping setup was as described previously [19]. Briefly, an NIR VSD, di-4-ANBDQBS, was used to record electrical signals from the heart. The dye was excited with collimated light using a 660 nm LED (M660L3, filtered at 650/40; from Thorlabs, USA). The emitted fluorescence was filtered with a 720 nm long-pass filter (NT46-066, Edmund Optics, USA), which was placed in front of a camera. Optical movies were obtained with a fast, 14-bit EMCCD camera (iXon^EM+^DU-860, Andor Technology, Ireland). The anterior surface and LV of the heart was imaged, and the field of view was 20 × 20 mm. Optical movies were acquired at a sampling rate of 500 Hz with a resolution of 128 × 128 pixels using imaging software (Andor SOLIS x-3467). The optical action potentials (OAPs) were taken from a 5 pixel × 5 pixel area. The background fluorescence (F) was subtracted from every frame of the recording. The optical signal was normalized with respect to the background fluorescence to obtain the fractional change in the fluorescence signal (Δ*F*/*F*).

### 2.6. Chemicals

CAR (≥97% purity) was purchased from Carl Roth GmbH + Co (Germany), (±)-blebbistatin was from Cayman (USA), and NIR VSD di-4-ANBDQBS and other chemicals were from Sigma-Aldrich (USA). Water-insoluble compounds were initially dissolved in dimethyl sulfoxide (DMSO) or ethanol to make stock solutions, which were then diluted. The highest concentration of the solvent was <0.1% and did not affect the measurements.

### 2.7. Data Analysis and Statistics

The AT was detected as the time interval from stimulus to 50% depolarization of the AP and OAP. The duration of the APD/OAPD was detected at the level of 20%, 50%, and 90% repolarization (APD20/OAPD20, APD50/OAPD50, and APD90/OAPD90, respectively) from AT. OAP maps were created from videos using custom the Scroll 1.16 software developed by Dr. S. Mironov (University of Michigan, USA).

Data are presented as the mean ± standard error of the mean (s.e.m.). The significance of differences was evaluated using one-way analysis of variance (ANOVA). The significance level was set at *p* < 0.05.

## 3. Results and Discussion

In earlier investigations, the role of CAR on the cardiovascular functions of animals has been studied *in vitro* and *in vivo*, and hypotensive or cardioprotective effects against acute myocardial infarction and ischaemia-reperfusion injury have been proposed [1, 14, 20, 21]. However, these studies were unable to reveal all details of the mechanism(s) of the CAR action occurring in a whole heart, such as the AP changes and the spread of electrical activity over the LV during different pacing conditions. This prompted us to investigate the ionic mechanisms underlying the effects of CAR, a known TRPM7 channel inhibitor [15], in a heart in detail.

**Figure 1:**
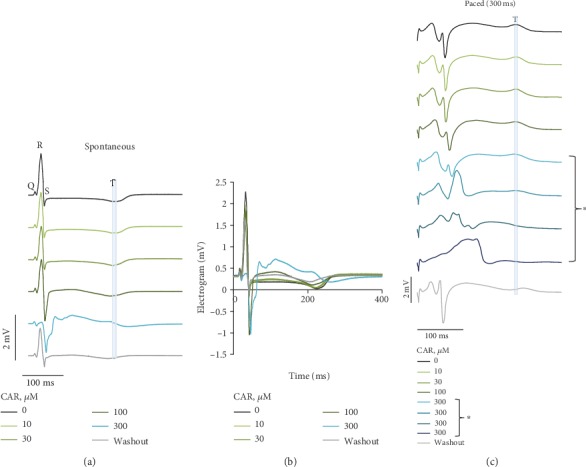
Representative traces of electrical activity registered on the Langendorff-perfused rabbit hearts. (a) Pseudo-ECGs under spontaneous rhythm in control conditions (black) and at 10, 30, 100, and 300 *μ*M cumulatively increased concentrations of CAR (light green, green, dark green, and light blue, respectively) for 5 min under each condition, followed by 10 min perfusion without the drug (grey). (b) Superimposition of pseudo-ECG traces; same data as in (a). (c) Traces obtained from a unipolar electrogram of the LV under the same experimental conditions as in (a) but during epicardial stimulation. Note: marked slowing in conduction velocity (CV) and depression in T-wave amplitude at 300 *μ*M CAR. Asterisk means electrogram traces presented in every 30 s (not after 5 min), when perfused with CAR at the 300 *μ*M concentration (light blue, blue, dark blue, and navy, respectively).

Initially, the effects of CAR on electrical recordings such as pseudo-ECGs (Figures 1(a) and 1(b)) and unipolar electrograms (EGs; Figure 1(c)) were studied in the Langendorff-perfused rabbit heart during spontaneous rhythm (almost equivalent to atrial stimulation) and under stimulation from the epicardial surface with a period of 300 ms.  Figure 1 shows typical examples of pseudo-ECG and EG traces during perfusion with the Tyrode solution (control) and cumulative perfusion with 10, 30, 100, and 300 *μ*M CAR followed by a washout period. The data show that the CAR at low concentrations of 10 and 30 *μ*M exerted no apparent effect on those electrical signals, measured during both spontaneous rhythm and epicardial stimulation. At higher concentrations, under both experimental conditions, CAR induced a decrease in conduction (widening in QRS), which resulted in marked slowing in the spread of electrical signals, especially at 300 *μ*M (Figures 1(a)–1(c), blue), and this is suggestive of impaired functioning of the Na^+^ channels. During epicardial stimulation (300 ms period), QRS widened from 59.08 ± 11.84 ms in control to 69.22 ± 13.43 ms with 100 *μ*M and to 78.44 ± 14.21 ms with 300 *μ*M CAR (*n* = 4, *p* < 0.05). In addition to a decrease in conduction, a depression in T-wave amplitude was observed on the EG (see solid vertical light blue line), likely suggesting impairment of the repolarization process as well. The QT interval under epicardial pacing was reduced from 234.62 ± 7.39 ms in control to 217.67 ± 11.09 ms with 100 *μ*M and to 208.92 ± 11.69 ms with 300 *μ*M CAR (*n* = 4, *p* < 0.05). Importantly, the effects were largely reversible upon washout (Figures 1(a)–1(c), grey).

After observing that CAR decreased electrical signal conduction, in our next step, we extended our studies to evaluate possible changes in various AP parameters under stimulation originating from different locations. We have previously demonstrated that when pacing was initiated via the atrial, endocardial, and epicardial surfaces, the spread of electrical activity varied, creating different situations in the myocardium [19]. Atrial pacing allows evaluation of the spread of electrical activity via the atrioventricular (AV) node.

**Figure 2:**
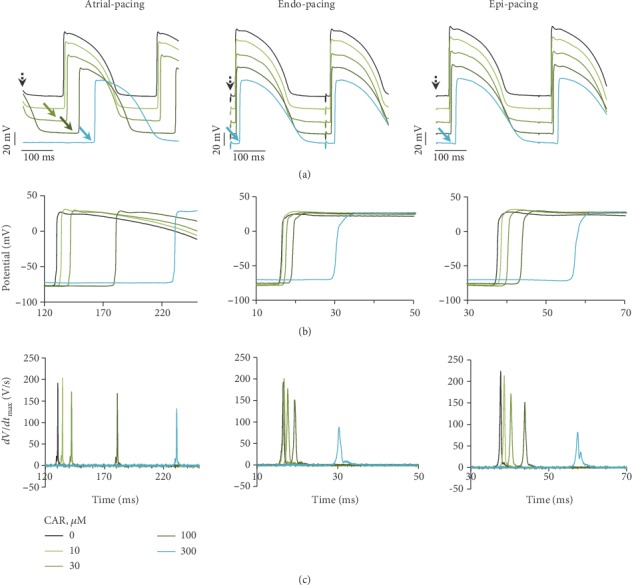
CAR effects on the APs of the Langendorff-perfused rabbit hearts. (a) Representative traces of APs from microelectrodes obtained with atrial, endocardial, and epicardial pacing under control conditions (i.e., Tyrode solution; black) and at 10, 30, 100, and 300 *μ*M cumulatively increased concentrations of CAR (light green, green, dark green and light blue, respectively). Insert: CAR concentrations as indicated in *μ*M. The broken black arrow indicates the start of the stimulus. Solid arrows denote the rightward shift in the AP activation time. (b) Superimposition of AP upstrokes from the same data as in (a). Note change in AP activation times vs. CAR concentrations as indicated. (c) Traces of *dV*/*dt*_max_ (in V/s) obtained at increased concentrations of CAR. AP upstroke velocity values were obtained from the same AP data as in (a). Note the marked decrease in conduction at 300 *μ*M CAR (light blue).

Figure 2(a) shows a typical example of the AP traces obtained with a standard glass microelectrode inserted in the LV wall from the epicardial surface of the rabbit heart under various concentrations of CAR when stimulation was applied from the atrium (left), endocardium (middle), and epicardium (right). Their superimposed upstrokes on an expanded time scale (Figure 2(b)) show activation time changes versus cumulative CAR concentrations in the three conditions. Note rightward shift of AP upstroke at high concentrations of CAR, possibly, due to marked slowing in conduction. In addition, Figure 2(c) shows that high concentrations of CAR (≥100 *μ*M), during atrial, endocardial, and epicardial pacing, induced significant decrease in the value of the maximal rate of the depolarisation of the APs upstroke (*dV*/*dt*_max_), which directly depends on the fast Na^+^ current (*I*_Na_). While at low concentrations of CAR (10-30 *μ*M), only negligible changes occurred (see Table 1). With exception, when stimulation was performed from the right atrium (Figure 2(c), left), then a significant (*p* < 0.05) increase in *dV*/*dt*_max_ magnitude was observed at 10 *μ*M, possibly because of an increase in the *I*_Na_ availability, which, we suppose, can be explained by reduction in APD.

The high concentrations of the drug (≥100 *μ*M) elicited a marked slowing in conduction; especially it is obvious under atrial stimulation. The microelectrode recordings under such experimental conditions show evidence of conduction abnormalities and demonstrate a 2 : 1 conduction block (not illustrated, but can be seen in Figure 2, i.e., the rightward shift of both APs and *dV*/*dt*_max_) under perfusion with CAR at 100 *μ*M (dark green) and 300 *μ*M (light blue) concentrations. Since cardiomyocytes of the AV node lack functional voltage-gated fast Na^+^ channels, when stimulated from the atrium, the AP in the AV node is formed mainly by the *I*_CaL_. So, a big delay observed under atrial pacing conditions can be explained by reduced Ca^2+^-dependent AV conduction. However, any modulation of *I*_CaL_ amplitude might be expected to induce parallel changes in contractile amplitude. Thus, obtained blocking action of CAR on the force of contraction (decrease by 30%, *n* = 4, *p* < 0.05) indirectly confirms inhibition of*I*_CaL_with the drug (see [Supplementary-material supplementary-material-1] in Supplementary Materials) and also contributes to the statement on reduction of Ca^2+^-dependent AV conduction under those experimental conditions.

Similarly, a significant decrease in the conduction velocity developed during endo- and epicardial pacing, but at higher (≥300 *μ*M) concentrations of CAR. However, under those experimental conditions, only the *I*_Na_ may have been responsible for conduction slowing. Consequently, decreased conductance with CAR could slow the conduction velocity both in the AV node and in the ventricular myocardium. The mean values of standard AP parameters calculated from five-to-nine experiments after CAR exposure (10-300 *μ*M) are presented in Table 1.

Additionally, at very high concentrations (≥300 *μ*M) of CAR, a significant decrease in overshoot potential was noticed (Figure 2(a), light blue), similarly as has been previously reported in canine ventricular myocytes with thymol, which is an isomer of CAR [22], probably, due to the blockage of Na^+^ channels. Besides, CAR and thymol inhibit compound action potentials in the frog sciatic nerve with almost the same affinity [23]. In contrast to low concentrations of CAR, superfusion with 1000 *μ*M CAR (not illustrated) induced rapid development of severe conduction abnormalities that was evident under all pacing types. This effect was accompanied by an enhanced stimulation threshold as well. However, all these changes were largely reversible upon washout of the drug.

In our study, there was no direct evidence that could be used to estimate the effects of CAR on the real amplitude of *I*_Na_. Nevertheless, the inhibitory effect of CAR on the *dV*/*dt*_max_ is sufficient confirmation of its action on the *I*_Na_ in the heart. Herein, our data demonstrating a decrease in the *dV*/*dt*_max_ to 47.7% and 38.7% (at 300 *μ*M) during endo- and epicardial pacing, respectively, agree with previous findings obtained for CAR in the dorsal root ganglia in which 300 *μ*M CAR reduced the peak *I*_Na_ by 47.8% [12].

**Figure 3:**
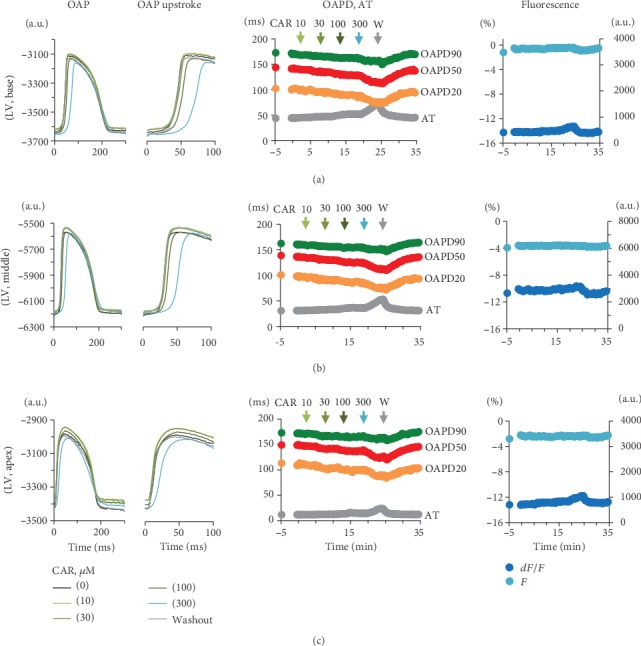
Effects of CAR on the OAPs of the Langendorff-perfused rabbit hearts. (a–c) Data obtained at the LV base, middle, and apex, respectively. Superimposition of OAPs and their upstrokes on an expanded time scale (left) of time-dependent changes in AT (grey) and the duration of OAPD20 (yellow), OAPD50 (red), and OAPD90 (green) (middle) of time-dependent changes of fluorescence in a.u. (*F*, light blue) and changes in the voltage-sensitive fraction of fluorescence in percent (*dF*/*F*, blue) versus time (right). The arrows above the graphs of OAPDs and AT (middle) indicate start of perfusion with corresponding concentration of CAR.

To extend our observations, we evaluated the effects of CAR on the optically recorded OAP parameters using a NIR VSD, di-4-ANDBQBS, on the LV of the rabbit hearts. Figures 3(a)–3(c) show a typical example of overlapped OAP recordings and OAP upstrokes on an expanded time scale as well as of changes in OAPD20, OAPD50, and OAPD90 (yellow, red, and green, respectively) and in AT (grey) as a function of time during perfusion with cumulatively increasing concentrations of CAR (10, 30, 100, and 300 *μ*M). The data obtained at three different locations in the LV, i.e., at a base, middle, and close-to-apex positions, are presented in Figures 3(a)–3(c), respectively, upon endo pacing (at 300 ms period). Again, at high concentrations (≥100 *μ*M) of CAR, the most prominent effect was a gradual decrease in the magnitudes of the OAP upstroke and OAPD, along with an increase in the AT. The different positions were chosen to verify the effects of CAR in the apex-to-base direction. In general, the upstroke of OAP was longer (Figures 3(a)–3(c), left) than that of the microelectrode AP (Figure 2(a)) because of the combined effect of the electrical signals obtained from multiple cells in the LV wall (from depth and spatial) [24]. However, different OAP upstroke morphologies (Figures 3(a)–3(c), left) seemingly occurred because of the varied propagation of electrical signals in the transmural and lateral directions [25]. Of note, the optical signal does not allow the real amplitude of the APs to be evaluated. Nevertheless, the fact that we can see a decline in the voltage-sensitive fraction of fluorescence expressed as a percentage at the highest concentrations of CAR, without a corresponding change in basal fluorescence (Figure 3, right, *dF*/*F* vs. *F*), indicates that the decline was due to the effect of CAR on the amplitude of the AP. The data presented in Figure 3 (middle) show that at cumulatively increasing concentrations of CAR, there was a progressive decrease in OAPD20 (yellow), OAPD50 (red), and OAPD90 (green) relative to the controls without CAR (note: control points started 10 min before the first concentration (10 *μ*M) of CAR was applied). In addition, a slowing in AT (light grey) was observed with concentrations ≥100 *μ*M CAR. It should be noted that steady-state levels of time-dependent changes in OAPDs under the cumulatively increasing concentrations of CAR were not reached (Figure 3, middle panels). It might be because the drug was added cumulatively, and the perfusion time at each CAR concentration was not long enough (5 min). In spite of this, the fact that after 10 min of washout the OAP parameters were recovered to the control level seemingly confirms that reduction was induced namely by CAR.

**Figure 4:**
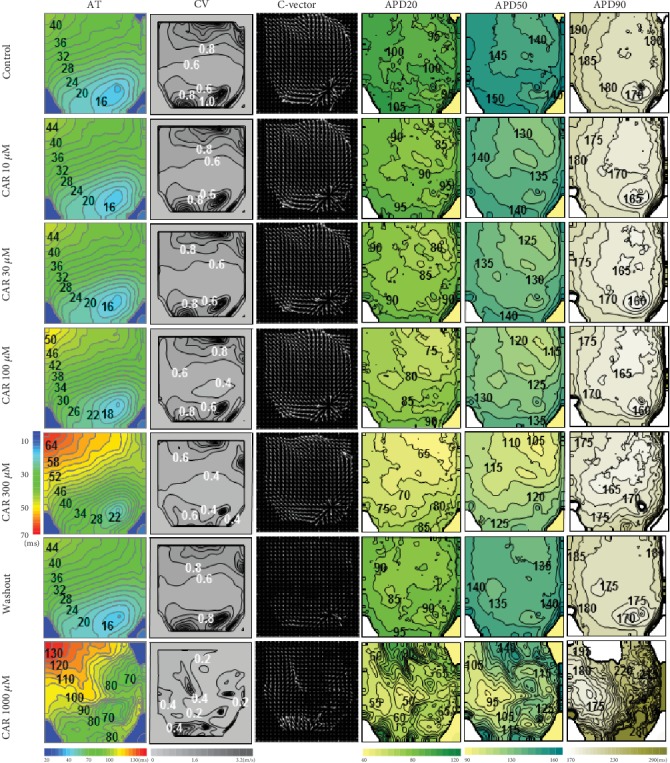
CAR effects on cardiac electrical activity of rabbit heart recorded by optical mapping using the fluorescent dye di-4-ANBDQBS. Representative optical maps of activation time (AT), conduction velocity (CV), conduction vector maps (C-vector), and OAPD maps (OAPD20, OAPD50, and OAPD90) obtained under control conditions and at 10, 30, 100, 300, and 1000 *μ*M concentrations of CAR. Endocardial pacing was set at 300 ms. The number near the isochrones shows the activation times in AT maps (in ms). The conduction velocity is shown in CV maps (in m/s). AP duration maps are shown for OAPD20, OAPD50, and OAPD90 (in ms). The interval between isochrones is 2 ms for the AT and 0.2 m/s for the CV. The AT was calculated as the time interval from stimulus to 50% depolarization. OAPD20, OAPD50, and OAPD90 were calculated at 20%, 50%, and 90% of repolarization, respectively, from the AT. Note: 1000 *μ*M CAR was applied after the washout period with Tyrode solution without the drug. The corresponding scale bars are given in the left and at the bottom.

To characterize the spatial and temporal dynamics of the OAP changes at cumulatively increasing concentrations of CAR, we constructed various maps: the activation time (AT), conduction velocity (CV), conduction vector (C-vector), OAPD20, OAPD50, and OAPD90 maps are presented in Figure 4 (from left to right, respectively) under the control and 10, 30, 100, 300, and 1000 *μ*M CAR treatments. Obviously, perfusion with high concentrations of CAR (≥300 *μ*M) seemingly resulted in detrimental damage at some cell layers of the LV, as evidenced by alterations in AT and conduction of the electrical signal, again due to the blockage of *I*_Na_, and these data are in coherence with both the AP and EG results. Nevertheless, all these effects were largely reversible upon washout. Of note, with 1000 *μ*M CAR, there was further decline at OAPD20 and OAPD50, whereas OAPD90 slightly lengthened relative to its duration with 300 *μ*M CAR. In some cells, this later lengthening could result in a longer OAP duration compared with the control, i.e., triangulation of APs might indicate serious proarrhythmia [26]. Again, all alterations were largely reversible upon washout.

The above data describe the effects of CAR on AP parameters in the Langendorff-perfused rabbit hearts from healthy animals. To date, human trials on the cardiovascular effects of CAR are still lacking, and we tested whether CAR could induce similar changes in a diseased heart, i.e., in the LV preparations dissected from the explanted hearts from patients who underwent heart transplantation surgery, which were provided by the hospital of LUHS for research purposes. We decided to test the effect of CAR at a 100 *μ*M concentration, based on a recent clinical trial in healthy volunteers that showed treatment with 1-2 mg/kg/day CAR (which is ~100 *μ*M in the blood) had no negative effects on pulmonary function and on the white blood cells [27].

**Figure 5:**
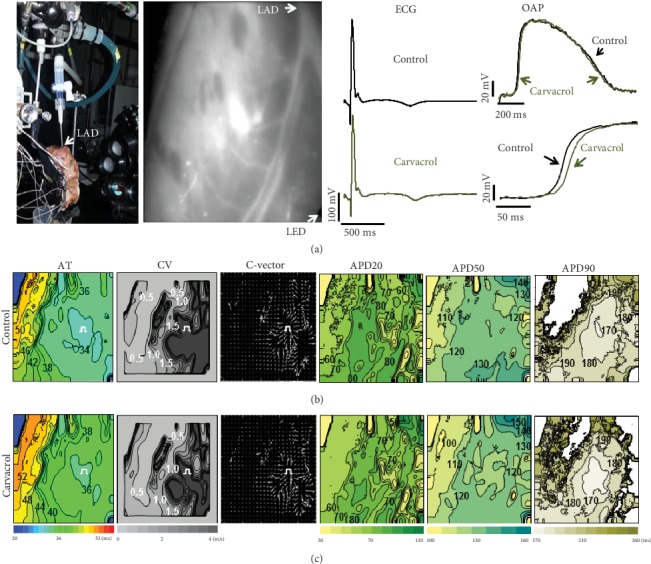
CAR effects on cardiac electrical activity in a human LV preparation obtained using the fluorescent dye di-4-ANBDQBS. (a) (left to right) schematic view of experimental setup, LV wedge preparation cannulated via left anterior descending coronary artery (LAD), ECG, superimposed OAPs (upper), and their upstrokes (lower); control (black) and 100 *μ*M CAR (green) treatments. Endocardial pacing was set at a 2000 ms period. Representative optical maps obtained during control (b) and 100 *μ*M CAR treatments (c): (left to right) activation time (AT), conduction velocity (CV), conduction vector (C-vector), and OAPD maps (OAPD20, OAPD50, and OAPD90). The corresponding scale bars are given at the bottom. Other notations are the same as in Figure 4.

Figure 5(a) shows an example (one out of three) of both ECGs and OAPs obtained using a di-4-ANBDQBS fluorescent dye under control conditions (black) and with CAR (100 *μ*M; green), with stimulation from the endocardial side (a period of 2000 ms). Examples of constructed maps for AT, CV, C-vector, OAPD20, OAPD50, and OAPD90 (from left to right, respectively) are presented in Figures 5(b) and 5(c) under the control and with CAR (100 *μ*M) treatment, respectively. The effects of CAR, as presented in Figure 5(a) (lower part), were quite similar to those obtained in the whole rabbit hearts (Figures 1(a) and 3(a)–3(c), on the left). In particular, there was a slight slowing in CV (from 0.66 ± 0.08 m/s to 0.55 ± 0.07 m/s, *p* = 0.049), lengthening in AT (from 140 ± 1.4 ms to 151.2 ± 1.6 ms, *p* = 0.003), rightward shifting in the OAP upstroke (Figure 5(a), lower part), and slight shortening of the OAPD (green). The mean values from three experiments before and after 5 min of perfusion with 100 *μ*M CAR were 304.4 ± 18.2 ms and 289.2 ± 19.0 ms for OAPD20 (*p* = 0.082), 494.0 ± 5.0 ms and 480.0 ± 5.5 ms for OAPD50 (*p* = 0.047), and 666.8 ± 4.8 ms and 652.0 ± 5.7 ms for OAPD90 (*p* = 0.037). These data show significant changes (*p* < 0.05), except for APD20, and indicate that CAR at 100 *μ*M concentration might affect the heart.

In general, in a human heart, resistance to pharmacological drugs, including CAR, might be higher than in hearts from quite young and healthy animals, possibly because of age-related changes and the medications used. However, considering that some OAP changes were significant, especially the AT lengthening, we suggest that the drug under certain circumstances might play a role in modulating cardiac electrical activity at concentrations ≥100 *μ*M.

The effects of CAR, which was previously identified as an effective TRPM7 channel inhibitor [15–17], on AP parameters in the Langendorff-perfused rabbit hearts and in human left ventricular preparations were comparable. Herein, our data clearly show that superfusion with high concentrations of CAR caused explicit modifications in various AP parameters; thus, different ion currents can retard/modify AP activation and repolarization. CAR actions include reduction in the *dV*/*dt*_max_ and overshoot potential, which directly depends on the fast *I*_Na_. While shortening of the APD (OAPD) depends on both *I*_CaL_ during the plateau (APD50) and *I*_K_, the total K^+^ current during the late phase of repolarization (APD90). CAR also altered the T-wave, an indirect index of repolarization, suggesting a defect in ventricular repolarization, and of potential participation of both *I*_CaL_ and *I*_K_ currents.

However, the TRPM7 channels might also contribute to these processes, especially under pathological conditions [16, 28–30]. The involvement of these channels in impaired cardiac automaticity and conduction defects has been implicated with a TRPM7 knock-in mouse model [31, 32]. In addition, TRPM7-like currents have been measured in ventricular cells of various animal species [33, 34] as well as in human atrial cardiomyocytes [16, 29, 30], and CAR, at a concentration of 100 *μ*M, almost completely blocked that current [16]. The TRPM7 channels are ubiquitous Mg^2+^ and Ca^2+^ permeable [35] and are thought to be responsible for Mg^2+^ transport into the cell at negative membrane potentials. The contribution of Mg^2+^ to the cardiac AP configuration is mainly important in the plateau (inhibits the inward movement of Ca^2+^ through the L-type Ca^2+^ channels, preventing Ca^2+^ overload) and late repolarization (inhibits the outward movement of K^+^ through the delayed rectifier K^+^ channels and blocks the inward rectifier K^+^ channels) phases. It should be noted that TRPM7-sensitive Ca^2+^ influx [36] could also facilitate an increase in the intracellular Ca^2+^ concentration.

Keeping in mind that cardiac electrical activity is often associated with an alteration in intracellular Mg^2+^, it is certainly important to determine the role of TRPM7 channels in the cardiac AP configuration. Such a contribution is currently unknown; obviously, in the absence of selective inhibitors for these channels, the real impact of the TRPM7-like current on the APD (or ECG) might be difficult to characterize even in the isolated cardiomyocytes. A study employing a transgenic TRPM7 knockout mouse model showed that the mechanisms responsible for TRPM7-mediated effects on cardiac electrophysiology are very complex [32]. Under physiological conditions, the Mg^2+^-sensitive TRPM7 current is not expected to alter the APD, given that the current is largely suppressed by physiological intracellular Mg^2+^. Nevertheless, the current might partly contribute to APD (or ECG) changes under the deficiency of Mg^2+^ ions and/or if the sensitivity to physiological intracellular Mg^2+^ decreased. In accordance with this assumption, the data obtained on Yorkshire swine left ventricular cardiomyocytes demonstrate that a decrease in cytosolic-free Mg^2+^ prolongs AP duration and could contribute to arrhythmogenesis in heart failure [37].

It is important to emphasize that CAR might not only bind to and thus modify the ion channel protein directly. However, as a hydrophobic compound, CAR can easily interfere with the lipid bilayer of the plasma membrane by increasing its fluidity and permeability to various ions, thus altering the local environment of ion channels [38, 39].

In summary, CAR is FDA-approved a dietary supplement and therefore is a safe compound to be tested in clinical research. Nevertheless, the data available in the literature to date regarding human trials on the cardiovascular effects of CAR are still lacking. Also, there is no demonstration of a pharmacological action of CAR on the whole heart preparations. The current study, we believe, provides valuable information about its action in the heart, which might be useful to study further and be translated into clinical research. Our key findings are as follows: Firstly, we demonstrate that mainly at high (≥100 *μ*M) concentrations CAR induces significant changes on electrical activity in the rabbit heart, when stimulated from the atrium, endocardium and epicardium. In particular, CAR increases QRS interval and affects the upstroke of AP by lowering the *dV*/*dt* and inducing a decrease in conduction velocity in the ventricular myocardium. These effects of CAR are undoubtedly due to the blocking of the *I*_Na_. Also, decrease of APD and modification of repolarization process were recorded what can be explained by alteration of *I*_Ca_ and *I*_K_. At higher (≥300 *μ*M) concentrations, CAR also provoked AV blockages and facilitated the development of an abnormal spread of electrical activity in the heart, additionally suggesting impaired functioning of the Na^+^ and Ca^2+^ channels. Importantly, these changes occurred without a significant impact on the resting membrane potential and were highly reversible. Secondly, of particular importance of this study is detailed translation from rabbit-to-human heart data. Here, on human ventricular wedges, using 100 *μ*M of CAR, similar changes on electrical activity were detected. Considering that some OAP changes were significant, especially the AT lengthening, we suggest that the drug under certain circumstances might play a role in modulating cardiac electrical activity at concentrations ≥100 *μ*M. However, it is important to mention that CAR has short half-life and is rapidly excreted by kidneys [40]. This highlights that special consideration should be for administration of CAR when translating to clinical studies. Finally, despite CAR was previously identified as an effective and relatively more specific antagonist of TRPM7 channel, herein, our data demonstrate that it is a nonspecific blocker. This might indicate that CAR, supposedly, will not allow detecting the real impact of TRPM7-like current on AP formation in the heart.

## 4. Conclusions

This study used CAR to assess its effect on electrical activity in the whole Langendorff-perfused rabbit heart and left ventricular wedges of the human heart. Our findings highlight that mainly high (≥100 *μ*M) concentrations of CAR elicited significant changes on electrical activity in the rabbit heart. In particular, a decrease in conduction velocity, especially during atrial pacing, causes delays in the normal conduction pathway. In addition, the data revealed that in human ventricle, similar changes on electrical activity might be detected with 100 *μ*M of the drug. Finally, despite CAR was previously identified as a relatively more specific antagonist of the TRPM7 channel, our data demonstrate that it is a nonspecific blocker as its action on Na^+^ and Ca^2+^ channels was revealed.

## Figures and Tables

**Table 1:** The mean data of standard AP parameters in a Langendorff-perfused rabbit heart.

Pacing	AT	*dV*/*dt*_max_	APA	APD20	APD50	APD90	RP
	(ms)	(V/s)	(mV)	(ms)	(ms)	(ms)	(mV)
Atrial							
Control	143.2 ± 6.4	164.2 ± 7.9	105.9 ± 1.1	91.7 ± 4.9	133.6 ± 3.7	162.1 ± 2.6	−81.0 ± 2.4
CAR10	147.7 ± 6.7^∗^	182.6 ± 8.1^∗^	107.0 ± 1.2	87.8 ± 3.5	129.2 ± 2.9^∗^	158.0 ± 2.6^∗^	−80.2 ± 0.9
CAR30	154.9 ± 6.3^∗^	149.9 ± 13.0	105.6 ± 1.2	87.6 ± 3.5	126.0 ± 3.4^∗^	155.2 ± 3.3^∗^	−79.4 ± 1.6
CAR100	172.4 ± 2.6^∗^	129 ± 14.7^∗^	103.8 ± 1.1^∗^	84.0 ± 4.5^∗^	121.4 ± 4.7^∗^	152.2 ± 3.6^∗^	−81.4 ± 1.9
CAR300	~226.9	~109.5	~97.9	~46.9	~77.7	~122	-78.4
Endo-							
Control	27.9 ± 1.9	161.0 ± 6.9	106.1 ± 1.2	89.2 ± 4.1	132.6 ± 2.9	161.5 ± 1.9	−82.8 ± 1.5
CAR10	29.1 ± 2.2	164.8 ± 7.9	107.1 ± 1.2	86.5 ± 3.7	128.7 ± 2.8^∗^	157.8 ± 1.8^∗^	−81.4 ± 0.9
CAR30	29.5 ± 2.1^∗^	150.5 ± 7.9	105.0 ± 1.4	85.5 ± 3.5	126.5 ± 2.7^∗^	156.0 ± 2.7^∗^	−80.4 ± 1.3
CAR100	31.1 ± 1.6^∗^	121.9 ± 8.9^∗^	100.8 ± 2.6^∗^	81.2 ± 4.4^∗^	120 ± 3.8^∗^	152 ± 2.3^∗^	−81.3 ± 1.3
CAR300	41.3 ± 3.2^∗^	76.9 ± 7.5^∗^	98.0 ± 2.3^∗^	72.1 ± 5.4^∗^	112 ± 6.6^∗^	154.8 ± 6.9	−78.6 ± 1.0
Epi-							
Control	41.6 ± 2.1	172.7 ± 10.0	106.3 ± 1.2	89.3 ± 3.9	131.1 ± 2.0	160.6 ± 2.2	−81.8 ± 2.4
CAR10	42.5 ± 2.5	169.7 ± 8.5	107.0 ± 1.2	89.1 ± 3.9	129.6 ± 3.3	158.2 ± 3.2	−80.5 ± 1.1
CAR30	44.1 ± 3.1	155.4 ± 8.1	105.2 ± 1.0	87.8 ± 3.2	125.4 ± 3.1^∗^	154.4 ± 2.9^∗^	−79.5 ± 1.6
CAR100	45.2 ± 2.9^∗^	124 ± 12.7^∗^	103.9 ± 1.0^∗^	83.7 ± 4.6^∗^	121.9 ± 5.1^∗^	153.1 ± 3.8^∗^	−80.7 ± 1.6
CAR300	62.8 ± 3.7^∗^	66.8 ± 11.8^∗^	97.9 ± 2.8^∗^	72.0 ± 5.4^∗^	111 ± 7.4^∗^	156.1 ± 7.3	−77.6 ± 0.8

Control: Tyrode solution containing blebbistatin (10 *μ*M); CAR10-300: carvacrol effects with the maximal changes in APD at 10 *μ*M, 30 *μ*M, 100 *μ*M, and 300 *μ*M CAR concentrations, respectively; RP: resting membrane potential; APA: action potential (AP) amplitude; APD20, APD50, and APD90: AP duration at 20%, 50%, and 90% levels of repolarization, respectively; *dV*/*dt*_max_: the maximal value of the first time derivative of the AP upstroke; AT: activation time. Statistical analysis was performed using ANOVA, ^∗^*p* < 0.05 for CAR vs. control, *n* = 5 for each during atrial and epicardial pacing, and *n* = 9 for each during endocardial pacing. Note that the data presented at 300 *μ*M CAR under atrial pacing are from one successful measurement. The mean ± standard error of the mean was not determined because of the appearance of atrioventricular blocks. The average depth of microelectrode insertion was 1.82 ± 0.57 mm from the epicardium.

## Data Availability

All data used to support the findings of this study are included within the article.

## Abbreviations

AP: Action potential
APA: Action potential amplitude
APD: Action potential duration
APD20, APD50, and APD90: APD at 20%, 50%, and 90% levels of repolarization
AT: Activation time
AV: Atrioventricular
CAR: Carvacrol (2-methyl-5-isopropylphenol)
di-4-ANBDQBS: A styryl voltage-sensitive fluorescence dye
F: Fluorescence
*dF*/*F*: Voltage-sensitive fraction of fluorescence in %
*dV*/*dt*_max_: The maximal value of the first time derivative of the AP upstroke
ECG: Electrocardiogram
EG: Electrogram
*I*_CaL_: L-type Ca^2+^ current
*I*_K_: K^+^ current
*I*_Na_: Voltage-gated Na^+^ current
NIR: Near-infrared
OAP: Optical action potential
TRPM7: Transient receptor potential melastatin 7 channel
VSD: Voltage-sensitive dye.

## Supplementary Materials

Supplementary Materials Figure S1: CAR effects on contractility in human right ventricular trabeculae. Superimposition of the individual contractile traces, which were obtained in control (black), after 10 min with 100 *μ*M CAR treatments (green), and during the washout period (grey) with normal Tyrode solution without the drug. Note: CAR significantly (*p* < 0.05) decreased force of contraction.

## References

[1] Yu W., Liu Q., Zhu S. (2013). Carvacrol protects against acute myocardial infarction of rats via anti-oxidative and anti-apoptotic pathways. *Biological & Pharmaceutical Bulletin*.

[2] Lima Mda S., Quintans-Júnior L. J., de Santana W. A., Martins Kaneto C., Pereira Soares M. B., Villarreal C. F. (2013). Anti-inflammatory effects of carvacrol: evidence for a key role of interleukin-10. *European Journal of Pharmacology*.

[3] Magi G., Marini E., Facinelli B. (2015). Antimicrobial activity of essential oils and carvacrol, and synergy of carvacrol and erythromycin, against clinical, erythromycin-resistant group A Streptococci. *Frontiers in Microbiology*.

[4] Guimarães A. G., Quintans J. S. S., Quintans-Júnior L. J. (2013). Monoterpenes with analgesic activity - a systematic review. *Phytotherapy Research*.

[5] Sharifi-Rad M., Varoni E. M., Iriti M. (2018). Carvacrol and human health: a comprehensive review. *Phytotherapy Research*.

[6] European Commission The register of flavouring substances annexed to this decision is hereby adopted. https://www.fsai.ie/uploadedFiles/Commission_Decision_1999_217_EC.pdf.

[7] EAFUS (2006). *A Food Additive Database. Centre for Food Safety and Applied Nutrition*.

[8] da Silva A. M. M., Empis J. M. A., Teixeira-Dias J. J. C. (2002). Inclusion of carvone enantiomers in cyclomaltoheptaose (*β*-cyclodextrin): thermal behaviour and H->D and D->H exchange. *Carbohydrate Research*.

[9] Guimarães A. G., Oliveira M. A., Alves Rdos S. (2015). Encapsulation of carvacrol, a monoterpene present in the essential oil of oregano, with beta-cyclodextrin: improves the pharmacological response on cancer pain experimental protocols. *Chemico-Biological Interactions*.

[10] Suntres Z. E., Coccimiglio J., Alipour M. (2015). The bioactivity and toxicological actions of carvacrol. *Critical Reviews in Food Science and Nutrition*.

[11] Günes-Bayir A., Kocyigit A., Güler E. M., Bilgin M. G., Ergün I. S., Dadak A. (2018). Effects of carvacrol on human fibroblasts (WS-1) and gastric adenocarcinoma (AGS) cells in vitro and on Wistar rats in vivo. *Molecular and Cellular Biochemistry*.

[12] Joca H. C., Vireira D. C. O., Vasconcelos A. P., Araújo D. A. M., Cruz J. S. (2015). Carvacrol modulates voltage-gated sodium channels kinetics in dorsal root ganglia. *European Journal of Pharmacology*.

[13] Magyar J., Szentandrássy N., Bányász T., Fülöp L., Varró A., Nánási P. P. (2004). Effects of terpenoid phenol derivatives on calcium current in canine and human ventricular cardiomyocytes. *European Journal of Pharmacology*.

[14] Dantas B. P. V., Alves Q. L., de Assis K. S. (2015). Participation of the TRP channel in the cardiovascular effects induced by carvacrol in normotensive rat. *Vascular Pharmacology*.

[15] Parnas M., Peters M., Dadon D. (2009). Carvacrol is a novel inhibitor of Drosophila TRPL and mammalian TRPM7 channels. *Cell Calcium*.

[16] Macianskiene R., Martisiene I., Zablockaite D., Gendviliene V. (2012). Characterization of Mg^2+^-regulated TRPM7-like current in human atrial myocytes. *Journal of Biomedical Science*.

[17] Chen W., Xu B., Xiao A. (2015). TRPM7 inhibitor carvacrol protects brain from neonatal hypoxic-ischemic injury. *Molecular Brain*.

[18] Xu H., Delling M., Jun J. C., Clapham D. E. (2006). Oregano, thyme and clove-derived flavors and skin sensitizers activate specific TRP channels. *Nature Neuroscience*.

[19] Mačianskienė R., Martišienė I., Navalinskas A. (2015). Evaluation of excitation propagation in the rabbit heart: optical mapping and transmural microelectrode recordings. *PLoS One*.

[20] Aydin Y., Kutlay Ö., Ari S., Duman S., Uzuner K., Aydin S. (2007). Hypotensive effects of carvacrol on the blood pressure of normotensive rats. *Planta Medica*.

[21] Chen Y., Ba L., Huang W. (2017). Role of carvacrol in cardioprotection against myocardial ischemia/reperfusion injury in rats through activation of MAPK/ERK and Akt/eNOS signaling pathways. *European Journal of Pharmacology*.

[22] Magyar J., Szentandrássy N., Bányász T., Fülöp L., Varró A., Nánási P. P. (2002). Effects of thymol on calcium and potassium currents in canine and human ventricular cardiomyocytes. *British Journal of Pharmacology*.

[23] Kawasaki H., Mizuta K., Fujita T., Kumamoto E. (2013). Inhibition by menthol and its related chemicals of compound action potentials in frog sciatic nerves. *Life Sciences*.

[24] Girouard S. D., Laurita K. R., Rosenbaum D. S. (1996). Unique Properties of cardiac action potentials recorded with voltage-sensitive dyes. *Journal of Cardiovascular Electrophysiology*.

[25] Hyatt C. J., Mironov S. F., Vetter F. J., Zemlin C. W., Pertsov A. M. (2005). Optical action potential upstroke morphology reveals near-surface transmural propagation direction. *Circulation Research*.

[26] Hondeghem L. M., Carlsson L., Duker G. (2001). Instability and triangulation of the action potential predict serious proarrhythmia, but action potential duration prolongation is antiarrhythmic. *Circulation*.

[27] Ghorani V., Boskabady M., Boskabady M. H. (2018). Effect of carvacrol on pulmonary function tests, and total and differential white blood cell counts in healthy volunteers: a randomized clinical trial. *The American Journal of Physiology*.

[28] du J., Xie J., Zhang Z. (2010). TRPM7-mediated Ca^2+^ signals confer fibrogenesis in human atrial fibrillation. *Circulation Research*.

[29] Zhang Y.-H., Sun H.-Y., Chen K.-H. (2012). Evidence for functional expression of TRPM7 channels in human atrial myocytes. *Basic Research in Cardiology*.

[30] Mačianskienė R., Almanaitytė M., Jekabsone A., Mubagwa K. (2017). Modulation of human cardiac TRPM7 current by extracellular acidic pH depends upon extracellular concentration of divalent cations. *PLoS One*.

[31] Sah R., Mesirca P., Van den Boogert M. (2013). Ion channel-kinase TRPM7 is required for maintaining cardiac automaticity. *Proceedings of the National Academy of Sciences of the United States of America*.

[32] Sah R., Mesirca P., Mason X. (2013). Timing of myocardial Trpm7 deletion during cardiogenesis variably disrupts adult ventricular function, conduction, and repolarization. *Circulation*.

[33] Gwanyanya A., Amuzescu B., Zakharov S. I. (2004). Magnesium-inhibited, TRPM6/7-like channel in cardiac myocytes: permeation of divalent cations and pH-mediated regulation. *The Journal of Physiology*.

[34] Tashiro M., Inoue H., Konishi M. (2014). Physiological pathway of magnesium influx in rat ventricular myocytes. *Biophysical Journal*.

[35] Nadler M. J. S., Hermosura M. C., Inabe K. (2001). LTRPC7 is a MgATP-regulated divalent cation channel required for cell viability. *Nature*.

[36] Massullo P., Sumoza-Toledo A., Bhagat H., Partida-Sanchez S. (2006). TRPM channels, calcium and redox sensors during innate immune responses. *Seminars in Cell & Developmental Biology*.

[37] Wei S.-K., Hanlon S. U., Haigney M. C. P. (2003). Beta-adrenergic stimulation of pig myocytes with decreased cytosolic free magnesium prolongs the action Potential and enhances triggered activity. *Journal of Cardiovascular Electrophysiology*.

[38] Ahmad A., Khan A., Akhtar F. (2011). Fungicidal activity of thymol and carvacrol by disrupting ergosterol biosynthesis and membrane integrity against Candida. *European Journal of Clinical Microbiology & Infectious Diseases*.

[39] Lambert R. J. W., Skandamis P. N., Coote P. J., Nychas G.-J. E. (2001). A study of the minimum inhibitory concentration and mode of action of oregano essential oil, thymol and carvacrol. *Journal of Applied Microbiology*.

[40] Tisserand R., Young R. (2013). *Essential oil safety: a guide for health care professionals*.

