# Supplementary material for: Effect of Carvacrol, TRP Channels Modulator, on Cardiac Electrical Activity
Source: Biomed Res Int. 2020 Apr 6;2020:6456805. doi: 10.1155/2020/6456805 (PMC7166271; doi:10.1155/2020/6456805)
Supplement: Supplementary Materials — Figure S1: CAR effects on contractility in human right ventricular trabeculae. Superimposition of the individual contractile traces, which were obtained in control (black), after 10 min with 100 μM CAR treatments (green), and during the washout period (grey) with normal Tyrode solution without the drug. Note: CAR significantly (p < 0.05) decreased force of contraction. [file 6456805.f1.pdf]

MS: #6456805

By: MantėAlmanaitytė, Jonas Jurevičius, and Regina Mačianskienė

Effect of carvacrol, TRP channels modulator, on cardiac electrical activity

Supplementary Materials

Effect of CAR on force of contraction of right ventricular trabeculae from explanted human heart

We also used human *trabeculae* isolated from the right ventricle, which was placed in an experimental chamber (1 ml volume) and perfused under constant flow (10 ml/min) at 37 ± 0.2 ˚C with an oxygenated (100% O_2) Tyrode solution (in mM: 135 NaCl, 5.4 KCl, 1.8 $CaCl_2$, 0.9 $MgCl_2$, 0.33 NaH_2PO_4, 10 glucose, and 10 HEPES; pH 7.4). *Trabeculae* were subjected to field stimulation with the following characteristics: stimulus pulse width was 2-ms, stimulation rate was 0.5 Hz, and amplitude was twice the diastolic threshold. Isometric contraction was recorded using a mechanoelectrical force isometric transducer (Harvard Aparatus). The CAR (100 µM) was applied after steady-state conditions were established, i.e after 60 min perfusion with Normal Tyrode solution.

It is well known, that a decrease in amplitude of I_{CaL} in the heart, might be expected to induce parallel decrease in force of contraction. Figure S1 shows an example of inhibition of force of contraction with the CAR (100 µM) in human *trabeculae* isolated from the right ventricle. The mean values from four experiments before and after 10 min of perfusion with CAR were 1.73 ± 0.19 mN and 1.20 ± 0.06 mN, respectively (p <0.05). These data provide some support to confirm that a decrease in I_{CaL}, due to CAR blocking action, might be related with a decrease in AV conduction and AP duration, when stimulated from the atrium, and which was discussed in the manuscript.

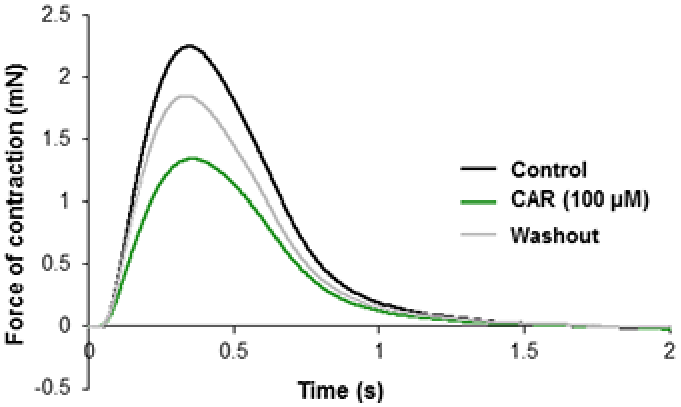

Figure S1. CAR effects on contractility in human right ventricular *trabeculae*. Superimposition of the individual contractile traces, which were obtained in control (*black*), after 10 min with 100 μM CAR treatments (*green*), and during the washout period (*grey*) with Normal Tyrode solution without the drug. Note: CAR significantly (p <0.05) decreased force of contraction.
